# Evidence-based surgery for laparoscopic cholecystectomy

**DOI:** 10.1016/j.sopen.2022.08.003

**Published:** 2022-08-18

**Authors:** Andrea T. Fisher, Kovi E. Bessoff, Rida I. Khan, Gavin C. Touponse, Maggie M.K. Yu, Advait A. Patil, Jeff Choi, Christopher D. Stave, Joseph D. Forrester

**Affiliations:** a Division of General Surgery, Department of Surgery, Stanford University, Stanford, CA; b University of California Berkeley, Berkeley, CA; c Stanford School of Medicine, Stanford University, Stanford, CA; d Department of Molecular, Cell, and Developmental Biology, University of California Los Angeles, Los Angeles, CA; e Department of Computer Science, Stanford University, Stanford, CA; f Lane Medical Library, Stanford School of Medicine, Stanford, CA

## Abstract

**Background:**

Laparoscopic cholecystectomy is frequently performed for acute cholecystitis and symptomatic cholelithiasis. Considerable variation in the execution of key steps of the operation remains. We conducted a systematic review of evidence regarding best practices for critical intraoperative steps for laparoscopic cholecystectomy.

**Methods:**

We identified 5 main intraoperative decision points in laparoscopic cholecystectomy: (1) number and position of laparoscopic ports; (2) identification of cystic artery and duct; (3) division of cystic artery and duct; (4) indications for subtotal cholecystectomy; and (5) retrieval of the gallbladder. PubMed, EMBASE, and Web of Science were queried for relevant studies. Randomized controlled trials and systematic reviews were included for analysis, and evidence quality was assessed using the Grading of Recommendations, Assessment, Development, and Evaluation framework.

**Results:**

Fifty-two articles were included. Although all port configurations were comparable from a safety standpoint, fewer ports sometimes resulted in improved cosmesis or decreased pain but longer operative times. The critical view of safety should be obtained for identification of the cystic duct and artery but may be obtained through fundus-first dissection and augmented with cholangiography or ultrasound. Insufficient evidence exists to compare harmonic-shear, clipless ligation against clip ligation of the cystic duct and artery. Stump closure during subtotal cholecystectomy may reduce rates of bile leak and reoperation. Use of retrieval bag for gallbladder extraction results in minimal benefit. Most studies were underpowered to detect differences in incidence of rare complications.

**Conclusion:**

Key operative steps of laparoscopic cholecystectomy should be informed by both compiled data and surgeon preference/patient considerations.

## INTRODUCTION

Approximately 10%–15% of American adults have cholelithiasis. Although the majority of these patients remain asymptomatic, roughly 1 in 5 will develop complications from their gallstones [1]. Development of minimally invasive cholecystectomy by French [2] and American surgeons in the late 1980s decreased the potential morbidity associated with cholecystectomy, leading to a broadening of indications for the procedure [1]. The Society of American Gastrointestinal and Endoscopic Surgeons (SAGES) identifies symptomatic cholelithiasis, biliary dyskinesia, acute cholecystitis, and complications of choledocholithiasis as indications for laparoscopic cholecystectomy in patients healthy enough to undergo the procedure [3]. Laparoscopic cholecystectomy (LC) is one of the most commonly performed surgeries, with 1.3 million of these procedures performed in the United States in 2021 [4].

Although several surgical societies have released LC guidelines including the SAGES expert Delphi consensus (2015) [5], SAGES guidelines for LC (2010) [3], European Association for Study of the Liver (EASL) LC guideline (2016), Tokyo guideline (2018), and World Society of Emergency Surgery (WSES) guideline (2020), considerable variation in the execution of key steps of the operation remains [6]. We hypothesized that evidence-informed standardization of key procedural steps would concur with expert recommendations and provide further guidance to encourage safe and efficient LC. This approach has been useful in the standardization of other common surgical procedures including cesarean delivery [7,8] and appendectomy [9]. This systematic review aims to critically evaluate evidence informing best practices for critical operative steps in laparoscopic cholecystectomy.

## METHODS

Research questions were framed using the population, indication, comparison, outcome (PICO) format. Based upon preliminary review of the literature, 5 main intraoperative decision points in laparoscopic cholecystectomy were identified by the authors (AF, KB, JF, JC): (1) number and position of laparoscopic ports; (2) identification of cystic artery and duct, including the use of selective intraoperative cholangiography; (3) division of cystic artery and duct; (4) subtotal cholecystectomy (SC) in difficult cholecystectomy; and (5) retrieval of the gallbladder. A research librarian (CS) worked with the team to generate comprehensive searches of PubMed (includes MEDLINE), EMBASE, and Web of Science for each PICO question (Supplementary File 1). The Preferred Reporting Items for Systematic Reviews and Meta-analyses guidelines were followed for identification and assessment of studies for inclusion. Search results were uploaded to Covidence systematic review software (Veritas Health Innovation, Melbourne, Australia; available at www.covidence.org). Three authors (AF, RK, and GT) screened abstracts relevant to each PICO question. We included English-language experimental, observational, and systematic review papers studying adults (age ≥ 18 years) undergoing laparoscopic cholecystectomy from database inception until April 29, 2021. We excluded case reports, animal studies, editorials, nonsystematic reviews, and nonapplicable studies. We also excluded society guidelines, although relevant guidelines are discussed and compared with findings in each section.

At least 3 randomized controlled trials (RCT) or systematic reviews were identified for each PICO question, and studies based on inferior evidence or performance (such as 2-day hospital stays after uncomplicated LC) were excluded. RCTs analyzed in systematic reviews for the same PICO question were not included separately. Disagreements were resolved by consensus. Data were extracted for each study using a standardized template including study type, comparators, primary and secondary outcomes, and quality of evidence. Evidence quality was assessed using the Grading of Recommendations, Assessment, Development, and Evaluation (GRADE) framework [10]. This study was prospectively registered with the PROSPERO database (registration: CRD42021225663). Because no individual patient information was collected, this study was exempt from IRB review.

## RESULTS

**Fig. 1:**
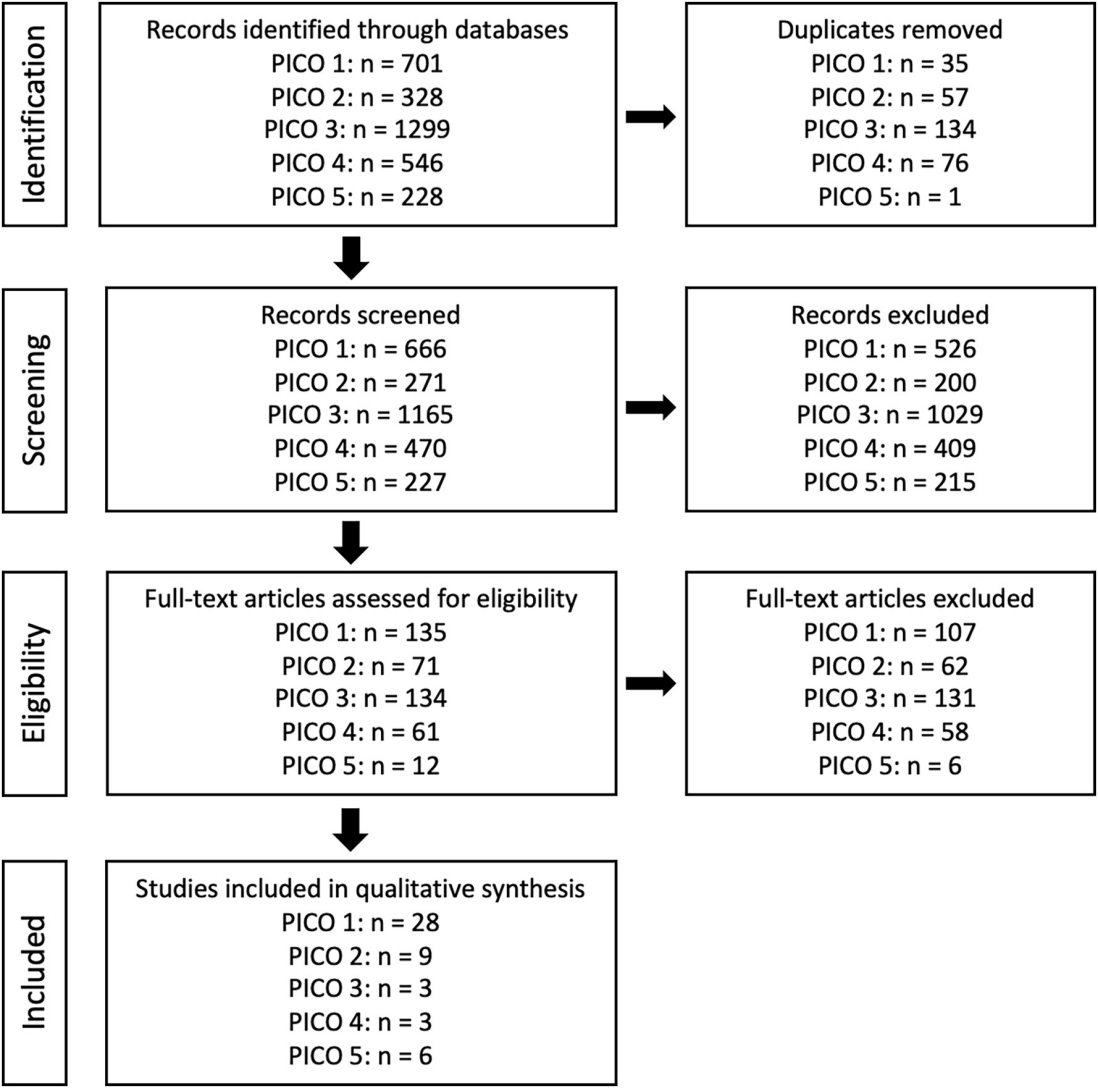
Articles reviewed for inclusion.

We identified 410 relevant studies from a preliminary review of 2,796 studies gathered through comprehensive searches of the literature. Full text review and elimination of redundant studies resulted in 49 studies available for analysis (See Fig. 1).

**PICO 1: In adult patients undergoing laparoscopic cholecystectomy (LC) for acute cholecystitis or symptomatic cholelithiasis, what is the best configuration of ports to limit perioperative morbidity (including port site hernia) and optimize surgical efficiency?**

### Background

The advent of single-incision LC, where up to 4 laparoscopic instruments are inserted via 1 umbilical incision, is representative of a trend to decrease the invasiveness of LC [11]. Reduced-port LC may result in improved cosmesis and reduced postoperative pain, although potential tradeoffs may include increased operative time [12,13]. Port closure technique, including sutured versus nonsutured fascial closure, must also be considered in the effort to minimize complications; this discussion is addressed in our group's previous publication [9]. Here, we evaluate 28 studies comparing standard 4-port laparoscopy (4-LC), 3-port laparoscopy (3-LC), 4-port mini-laparoscopy (MLC), 2-port laparoscopy (2-LC), and single incision laparoscopic cholecystectomy (SILC) (Table 1). Studies evaluating transvaginal or natural orifice transluminal endoscopic surgery were not compared.

**Table 1:** Port placement techniques

*Port technique*	*Abbreviation*	*Description*	*Number of studies*
Four-port laparoscopic cholecystectomy	4-LC	One 10-mm optic trocar, three 5-mm working trocars	21
Three-port laparoscopic cholecystectomy	3-LC	One 10-mm optic trocar, two 5-mm working trocars	10
Two-port laparoscopic cholecystectomy	2-LC	Two incisions house 2–3 trocars. Needlescopic instruments without trocars may be inserted elsewhere. Retention sutures may be placed.	5
Single-incision laparoscopic cholecystectomy	SILC	All instruments through an incision at the umbilicus using specialized SILS multiport device OR multiple trocars in same incision	16
Four-port mini-laparoscopic cholecystectomy	MLC	One 5- or 10-mm optic trocar and three 3-mm "needlescopic" working trocars	6

### Reduced-Port LC (2-LC, 3-LC)

Six RCTs and 2 systematic reviews compared 2- and 3-port LC against 4-port LC [[12], [13], [14], [15], [16], [17], [18], [19]] (Table 2). Two-incision approach differed slightly between studies: Poon et al used a modified operating telescope through 1 of the ports [16], Tavassoli et al used two 5-mm ports inserted into the umbilical incision and one 10-mm port in the hypogastrium [17], and Sreenivas et al used 2 ports and 2 additional needlescopic transabdominal graspers [18].

**Table 2:** 2-LC and 3-LC versus 4-LC

*Series*	*Type*	*Setting/Studies*	*Patients/Study inclusion criteria*	*2- or 3-LC (*N*)*	*4-LC (*N*)*	*Outcomes assessed*	*Conclusion*	*Quality of evidence*	*GRADE recommendation:*
Chohan et al	RCT	Single center	Included chronic cholecystitis/biliary colic Excluded acute cholecystitis, choledocholithiasis, cirrhosis	50 (3-LC)	50	24-h postop pain Complications Length of operation	For 3-port: Less postop pain (2.44 vs 4.52, *P <* .0001) No difference in operative time, length of stay, conversion to open, or complications.	2B	Three-port LC may result in less short-term postoperative pain than 4-port LC without increasing operative time.
Liu et al	RCT	Single center	Included elective LC Excluded gallstone pancreatitis, cholangiocarcinoma, choledocholithiasis, previous abdominal surgery	110 (3-LC)	106	Postop pain Length of stay Length of operation Days before return to work Cosmesis and quality of life at 3 mo	For 3-port: Less postop pain (2.3 ± 2.1 vs 4.3 ± 2.6, *P <* .01) Shorter length of stay (2.2 ± 1.5 vs 3.6 ± 1.7 d, *P <* .05) Faster return to work (5.3 ± 2.7 vs 7.8 ± 2.5 d, *P <* .05) Better cosmesis (90% vs 57% satisfaction) No difference in operative time	2A	Three-port LC is associated with improved postoperative pain, shorter time to discharge and resumption of activity, and better cosmesis and patient satisfaction after 3 mo without differences in operative time.
Singal et al	RCT	Single center	Included symptomatic cholelithiasis Excluded acute cholecystitis	100 (3-LC)	100	Postop pain and analgesia Operative time Complications	For 3-port: Less postop pain (83% vs 97% requiring analgesic) Longer operative time (93.16 vs 50.66 min) No difference in length of stay	Inadequate data to support a recommendation	This study does not provide adequate data to support a recommendation for 3- or 4-port LC—no *P* values were reported.
Poon et al	RCT	Single center	Included elective LC Excluded ASA III/IV and INR > 1.5	58 (2-LC)	57	Postop pain Analgesia use Operative time Length of stay	For 2-incision group: Shorter operative time (54.6 ± 24.7 min vs 66.0 ± 32.6 min, *P =* .04) No differences in pain, length of stay, or complications	2A	Two-port LC is associated with equivalent postoperative pain scores and complication rate compared with 4-port LC, without an increase in operative time.
Tavassoli et al	RCT	Single center	Included symptomatic cholelithiasis. Excluded acute cholecystitis, cholangitis, choledocholithiasis, and previous abdominal surgery.	70 (2-LC)	70	Postop pain Operative time Time to return to work Length of hospital stay Cosmesis	For 2-incision group: Lower pain (0.54 ± 0.86 vs 2.47 ± 1.71, *P <* .0001) Quicker return to work (3.37 ± 1.49 d vs 4.94 ± 1.31 d, *P <* .0001) Better cosmesis (9.71 ± 0.45 vs 8.03 ± 1.38, *P <* .0001) Shorter hospital stay (0.92 ± 0.31 vs 2.24 ± 0.95, *P <* .0001) No differences in operative time or complications	2A	Two-incision LC is associated with reduced pain, improved cosmesis, and quicker return to baseline compared with 4-port LC, without increases in operative time or complication rates.
Sreenivas et al	RCT	Single center	Included symptomatic cholelithiasis. Excluded acute cholecystitis, cholangitis, choledocholithiasis, and previous abdominal surgery.	55 (2-LC)	48	Postop pain Analgesia Operative time Length of hospital stay Time to return to baseline Cosmesis at 30 d	For 2-incision group: Improved pain up to 24 h at each time point Fewer analgesia doses (2.31 ± 1.01 vs 2.85 ± 0.79, *P =* .003) Improved cosmesis (7.55 ± 1.28 vs 5.90 ± 0.83, *P =* .001) Quicker return to baseline (4.25 ± 1.29 vs 5.17 ± 1.22 d, *P =* .001) No differences in length of stay, operative time, or complications	2A	Two-port LC with additional needlescopic graspers is associated with decreased immediate postoperative pain, better cosmesis, and quicker recovery compared with conventional 4-port LC, without increases in operative time or complications.
Gurusamy et al	Systematic review	9 RCTs	RCTs comparing fewer-than-4-port LC (SILC, 2-port, 3-port) to 4-port LC	427 (fewer-than-4 ports)	428	Return to activity Return to work Operative time Length of stay Cosmesis Quality of life	For less-than-4-port LC: Quicker return to activity (4.9 vs 6.1 d, *P <* .05) and return to work (10 vs 12 d, *P <* .05) Longer operative time (70.44 vs 56 min, *P <* .05) No difference in length of stay, quality of life, cosmesis, or complications	2A	Fewer-than-4-port LC may result in longer operative times but quicker return to baseline. No differences in safety were detected, but benefits of reduced port LC were too limited to recommend it over 4-port LC.
Hajibandeh et al	Systematic review	12 RCT, 5 observational studies	Included RCTs and cohort studies comparing 3-port versus 4-port	477 RCT, 601 obs (3-LC)	484 RCT, 549 obs (3-LC)	Postop pain at 12 and 24 h Operative time Conversions/complications Length of stay Return to baseline	For 3-port: Less postop pain (mean difference − 0.66 at 12 h, *P <* .00001 and − 0.54 at 24 h, *P <* .00001) Quicker return to baseline (mean difference − 0.79 d, *P =* .02) No difference in operative time, conversion rate, complications, or length of stay.	1A	Three-port LC is associated with less short-term postoperative pain and quicker return to activity compared with 4-port LC without differences in complication rates or operative time.

All 4 studies comparing 3-LC and 4-LC demonstrated less postoperative pain and quicker return to work/physiologic baseline among 3-LC patients [[12], [13], [14], [15]]. For 2-LC versus 4-LC, Tavassoli and Sreenivas found decreased pain and quicker return to baseline after 2-LC [17,18], whereas Poon did not find improvements in pain [16]. Poon et al notably conducted careful patient blinding with application of surgical dressings at four sites for both groups and adequately powered the study to detect 30% reduction in pain score. Gurusamy et al did not investigate pain in their meta-analysis of 4-LC versus 2-LC and 3-LC but did report quicker return to baseline in the reduced-port LC group [19].

All RCTs investigating cosmesis saw improvement with reduced-port LC. Liu et al found higher cosmetic satisfaction at 3 months among the 3-LC group with 90% reporting high satisfaction with their scars vs 57% of the 4-LC group, although they did not report a *P* value [12]. Tavassoli et al [17] and Sreenivas et al [18] detected significantly improved cosmesis for the 2-LC group (Table 2). However, one systematic review investigated cosmesis and determined that reduced-port LC did not produce significantly better cosmetic results than 4-LC [19].

Only Singal et al found increased operative time for reduced-port LC (93.16 min for 3-LC vs 50.66 min for 4-LC), although this study suffered from a lack of *P* values [13]. Poon et al even found significantly lower operative time for 2-LC (54.6 min vs 66.0 min, *P* = .04) [16]. However, it should be noted that for use of unique 2-LC equipment and techniques, operative time and safety depend heavily on surgeon familiarity.

No articles, including Hajibandeh's meta-analysis of 2,111 patients [14] and Gurusamy's meta-analysis of 855 patients, demonstrated increased complications for 2-LC or 3-LC when compared with 4-LC. Several studies had significant statistical issues: Singal et al did not report *P* values, and [13] Liu et al did not report power calculations [12]. All RCTs were underpowered to detect differences in complication rates. Gurusamy et al reported that most trials included in their systematic review were at high risk of bias, and authors did not feel that they could issue a recommendation given the current base of evidence [19]. Compared with 4-LC, 2-LC and 3-LC may result in quicker return to baseline without significant safety concerns, while evidence for pain reduction and improved cosmetic satisfaction remains conflicted.

### Mini-Laparoscopic LC

Five RCTs compared 4-port mini-laparoscopic cholecystectomy (MLC) to conventional 4-LC [[20], [21], [22], [23], [24]] (Table 3). Huang et al [22] also included an additional group undergoing LC with all 5-mm ports. Two studies, Alhashemi et al and Bisgaard et al, were stopped early because of technical issues (instrument breakage and instrument malfunctions, respectively) necessitating trocar upsizing [20,24]. All remaining studies demonstrated postoperative pain reduction of varying degrees among patients undergoing MLC. Bignell and Novitsky observed improved cosmesis after MLC [21,23], whereas Huang saw no cosmetic improvement after MLC compared with conventional LC or LC with all 5-mm ports [22]. Huang found that MLC resulted in longer operative time [22], whereas Bignell and Novitsky did not [21,23]. None of the RCTs that were completed reported differences in complication rates between groups. Although there were some consistent benefits to MLC including less pain, the fact that 2/5 RCTs were unsuccessful because of instrument issues must be considered by any surgeon contemplating adding MLC to their repertoire. The 3 completed studies either were powered to detect differences in pain/cosmesis rather than complications [21,23] or did not have power calculations provided [22]. The technical difficulty of switching to MLC may outweigh the benefit of mild pain reduction.

**Table 3:** 4-LC versus MLC

*Series*	*Type*	*Setting/Studies*	*Patients/Study inclusion criteria*	*4-LC (*N*)*	*MLC (*N*)*	*Other comparators*	*Outcomes assessed*	*Conclusion*	*Quality of evidence*	*GRADE recommendation:*
Alhashemi et al	RCT	Single center	Included patients undergoing elective LC Excluded acute cholecystitis	42	33		Postop pain at 1 and 3 mo Cosmesis and fatigue at 1 mo postop	17 MLC required upsizing to at least one 5-mm port versus 1 CLC conversion to open, study terminated early For MLC: Better cosmesis (mean difference 0.5 units at 1 mo, *P* = .009 and 1.0 units at 3 mo, *P =* .02) Postop pain lower at 3 mo No difference in activity levels	Inadequate data to support a recommendation	This study does not provide adequate data to support a recommendation for MLC versus CLC (study terminated early).
Bignell et al	RCT	Single center	Included elective and day case LC Excluded acute cholecystitis	40	40		Postop pain at 6 h Cosmesis at 6 mo	For MLC: Less pain (2.5 ± 2.1 vs 4.2 ± 2.9, *P =* .003 at 1 h and 0.8 ± 2.2 vs 2.1 ± 2.4, *P =* .002 at 1 wk) Better cosmesis (90% vs 35% with high satisfaction) No differences in operative time or complications	2A	MLC may result in lower postoperative pain and improved patient satisfaction with cosmetic outcome compared with CLC.
Huang et al	RCT	Single center	Included symptomatic cholelithiasis	30	30	All 5-mm port group (*n* = 30)	Postop pain at 24 h Cosmesis Length of stay Operative time Analgesia use	For MLC: Lower subxiphoid pain score at 24 h (CLC 4.7 ± 2.5, 5-mm LC 6.5 ± 3.1, MLC 5.4 ± 3.2, *P =* .02) Longer operative time (CLC 47.3 ± 20.8 min, 5-mm LC 49.8 ± 20.8 min, MLC 64.8 ± 27.7, *P =* .03) No difference in cosmesis, length of stay, or complications	2B	MLC may require longer operative times compared to conventional LC without significant differences in postoperative pain or cosmesis.
Novitsky et al	RCT	Single center	Included symptomatic cholelithiasis Excluded acute chole, age > 70, previous abdominal surgery, ASA III/IV, liver or coagulation disorder	33	34		Postop pain at days 1–28 Cosmesis at 1 mo	8 MLC conversions to CLC and excluded. For MLC: Lower postop pain on day 1 only (3.6 ± 1.5 vs 4.9 ± 1.8, *P =* .04), no differences days 3–28 Better cosmesis (38.9 ± 2.1 vs 28.9 ± 5.7, *P <* .001) No differences in complications or operative time	2B	MLC may result in lower immediate postoperative pain and better cosmesis than CLC without significant differences in operative time.
Bisgaard et al	RCT	Single center	Included symptomatic cholelithiasis Excluded s/p ERCP, ASA III/IV, chronic pain	13	13		Postop pain 0–3 h Operative time	Stopped early because of 5/13 MLC conversions. For MLC: Longer operative time (85 vs 55 min, *P =* .016) Postop pain less for some measures	Inadequate data to support a recommendation	This study does not provide adequate data to support a recommendation for MLC versus CLC (study terminated early).

### Single-Incision LC

Twelve RCTs and 3 systematic reviews compared SILC with greater port number LC [[25], [26], [27], [28], [29], [30], [31], [32], [33], [34], [35], [36], [37], [38], [39]]. Of the 6 RCTs comparing SILC against 4-LC (Table 4), 4 concluded that SILC was associated with less postoperative pain [25,26,28,30]. A systematic review by Tamimi et al (SILC versus 3-LC or 4-LC) showed similar results, with improved postoperative pain at 24 hours, shorter length of hospital stay, and quicker return to baseline after SILC [38]. Umemura et al compared SILC against MLC, discovering that SILC patients had lower pain at 24 hours and required fewer doses of analgesia [37]. However, postoperative pain differed substantially among studies comparing SILC and 3-LC (Table 5): one study saw increased postoperative pain for patients who underwent SILC [33], another saw decreased postoperative pain after SILC [34], and one observed no difference between groups [32]. Justo-Janeiro et al (SILC versus 2-LC versus 3-LC) noted less immediate postoperative pain in the SILC group but more pain at their final time point of 8 days [35].

**Table 4:** SILC versus 4-LC

*Series*	*Type*	*Setting/Studies*	*Patients/Study inclusion criteria*	*SILC (*N*)*	*4-LC (*N*)*	*Outcomes assessed*	*Conclusion*	*Quality of evidence*	*GRADE recommendation*
Bresadola et al	RCT	Single center	Included elective LC, ASA I/II Excluded acute cholecystitis	45	45	Postop pain Analgesia use Operative time Length of hospital stay	28% of patients were excluded for logistical and technical reasons For SILC: Less pain (*P <* .01) and analgesia use (88 ± 39 vs 113 ± 14 mg, *P <* .05) in first 24 h postop No differences in operative time, complications, or length of hospital stay 13/45 single incision group required extra trocars	2B	SILC may result in less postoperative pain compared with 4-port LC.
Chang et al	RCT	Single center	Included elective LC, ASA I/II Excluded acute cholecystitis, previous abdominal surgery	50	50	Postop pain at 4 h, 24 h, 2 wk, 6 mo Analgesia use Time to return to baseline Cosmesis at 2 wk and 6 mo	For SILC: Reduced pain at 24 h at extraumbilical sites (0.628 ± 1.394 vs 1.898 ± 2.617, *P =* .004) Longer operative time (79.46 vs 58.88 min, *P =* .003) No differences in complications, analgesia use, pain at other time points, time to return to baseline, or cosmesis 3/50 SILC conversion to conventional	2A	SILC may result in less postoperative pain compared with 4-port LC without significant improvement in patient cosmetic satisfaction. SILC and 4-port LC are roughly comparable from a safety perspective.
Goel et al	RCT	Single center	Included symptomatic cholelithiasis Excluded acute cholecystitis and gallbladder carcinoma	30	30	Postop pain Operative time Length of stay Cosmesis at 6 and 12 wk	For SILC: Longer operative time (64.6 vs 48.3 min, *P <* .05) Higher complication rates (bile spillage, bleeding, and difficult extraction, *P <* .05) Better cosmesis (*P <* .05) No differences in length of stay, postop pain, or conversion to open rate	2B	SILC may result in better cosmesis but longer operative times and higher rates of certain intraoperative difficulties.
Vilallonga et al	RCT	Multicenter	Included symptomatic cholelithiasis	69	71	Postop pain at 12 h Operative time Length of stay Cosmesis at 3 mo	For SILC: Lower postop pain (2.0 ± 0.8 vs 2.9 ± 1.2, *P <* .001) Longer hospital stay (38.5 ± 21.8 vs 24.1 ± 16.6 h, *P <* .001) Improved cosmesis (8.8 ± 0.9 vs 7.5 ± 1.3, *P <* .001) No difference in operative time or complication rates	2A	SILC may result in less immediate postoperative pain and higher cosmetic satisfaction without increased complications or operative time.
Lurje et al	RCT	Multicenter	Included symptomatic cholelithiasis Excluded pregnancy, coagulopathy, cirrhosis, taking DAPT	55	55	Postop pain Analgesia use Operative time Length of stay Cosmesis	For SILC: Lower postop pain on day 2 (1.0 ± 1.0 vs 2.0 ± 2.0, *P =* .001) and day 7 (1.0 ± 1.0 vs 2.0 ± 2.0, *P =* .005) Increased operative time (101 ± 36 vs 90 ± 41 min, *P =* .031) Improved cosmesis at 12 wk (21 vs 16, *P <* .001) and 1 y (24 vs 16, *P <* .001) No differences in complications or length of stay	2A	SILC results in better cosmesis and less postoperative pain but longer operative times than 4-port LC. Its safety profile is equivalent.
Subirana et al	RCT	Single center	Included symptomatic cholelithiasis, ASA I/II Excluded acute chole, BMI > 35, Mirizzi syndrome, choledocholithiasis, previous abdominal surgery, bleeding disorders	37	36	Postop pain Operative time Time to return to activity/work Cosmesis at 30 d Surgeon-rated difficulty	For SILC: Greater subjective difficulty (3.17 vs 1.94, *P =* .027) Better cosmesis (9.86 ± 0.58 vs 7.78 ± 1.50, *P <* .001) Fewer patients taking > 2 wk to return to work (7 vs 14, *P =* .014) No differences in postop pain, operative time, complications, or return to normal activity.	2A	SILC may be more technically difficult but may result in improved cosmesis without differences in safety or operative time.
Allemann et al	Systematic review	11 RCTs, 60 observational	RCTs and observational studies reporting BDI during SILC	438 RCT	401 RCT, 3599 obs	BDI Overall biliary complications	Nonsignificant increased risk of BDI (0.4% vs 0%, *P =* .36) and biliary complications (1.6% vs 0.5%, *P =* .21)	2A	SILC was not associated with significantly higher rates of biliary complications.

**Table 5:** SILC versus 3-LC

*Series*	*Type*	*Setting/Studies*	*Patients/Study inclusion criteria*	*SILC (*N*)*	*3-LC (*N*)*	*Outcomes assessed*	*Conclusion*	*Quality of evidence*	*GRADE recommendation*
Omar et al	RCT	Single center	Included symptomatic cholelithiasis, ASA I/II/III Excluded choledocholithiasis, Mirizzi syndrome, cholangiocarcinoma, previous abdominal surgery	89	98	Postop pain at 6 h and 24 h Operative time Cosmesis at 1 mo	For SILC: Increased operative time (58.9 ± 18.6 vs 45.2 ± 11.8 min, *P =* .001) Improved cosmesis (7.9 ± 1.6 vs 6.7 ± 1.4, *P =* .008) No difference in postop pain or complications 7 SILC patients needed extra port, 1 conversion to open versus 1 three-port LC conversion to open	2A	SILC results in better cosmesis but longer operative time, without differences in complication rates or postoperative pain.
Deveci et al	RCT	Single center	Included symptomatic cholelithiasis, ASA I/II/III Excluded choledocholithiasis, pregnancy, peritoneal dialysis, previous abdominal surgery, pancreatitis	44	42	Postop pain at 24 h Operative time Length of hospital stay Cosmesis at 6 mo	For SILC: Increased postop pain (3.32 ± 1.18 vs 2.32 ± .97, *P <* .001) Longer operative time (73 ± 32.7 vs 48 ± 15.1 min, *P <* .001) Improved cosmesis (4.28 ± 1.06 vs 3.30 ± 0.93, *P <* .001) No differences in length of stay or complications	2A	SILC may require longer operative time but may result in better cosmetic outcomes.
Pan et al	RCT	Single center	Included symptomatic cholelithiasis, ASA I/II/III Excluded acute cholecystitis, choledocholithiasis, Mirizzi syndrome, cholangiocarcinoma, previous abdominal surgery	49	53	Postop pain at 8 h and 7 d Analgesia Blood loss Operative time Length of hospital stay Cosmesis at 2 mo	For SILC: Less postop pain (2.0 ± 1.5 vs 3.6 ± 1.6, *P <* .0001) Higher cosmesis scores (8 ± 0.4 vs 6 ± 0.2, *P <* .0001) No differences in operative time, length of hospital stay, complication rates, or postop pain at day 7	2A	SILC may result in lower immediate postoperative pain and improved cosmesis without significant increases in operative time.

Four of 6 RCTs comparing SILC and 4-LC and all 3 studies comparing SILC and 3-LC recorded improved cosmesis scores with SILC [[27], [28], [29], [30],[32], [33], [34]]. Umemura et al saw no difference in cosmetic satisfaction at 3 and 14 days postoperatively but failed to track cosmetic satisfaction as long as other studies [37]. Tamini et al performed the only systematic review to investigate cosmesis and found improvement after SILC compared with 3- or 4-LC [38].

Operative time was consistently longer for SILC. In 6 RCTs [[26], [27], [28],^32^,^33^,^35^] and the 2 systematic reviews that investigated operative time [38,39], SILC took longer to complete than 3-LC, 4-LC, or MLC (Table 6). Although Subirana et al did not find a difference in operative time, they noted that surgeon-rated subjective difficulty was higher for SILC [29]. Sulu et al specifically sought to determine whether operative duration for SILC could be reduced by adding a sub-xiphoid port for gallbladder retraction and noticed that operative time was halved for 2-LC compared with regular SILC (35.0 ± 12.3 vs 79.1 ± 7.7 min, *P* < .05) [36] (Table 5).

**Table 6:** Other PICO 1 studies

*Series*	*Type*	*Setting/Studies*	*Patients/Study inclusion criteria*	*SILC (*N*)*	*2-LC (*N*)*	*3-LC (*N*)*	*MLC (*N*)*	*Other comparators*	*Outcomes assessed*	*Conclusion*	*Quality of evidence*	*GRADE recommendation*
Justo-Janeiro et al	RCT	Single center	Included elective LC, ASA I/II	18	18	19			Postop pain Analgesia Operative time Length of hospital stay	For SILC: Less immediate postop pain (*P =* .02), more pain at 8 d (*P =* .03) Longer operative time (67 ± 21.9 min vs 54.7 ± 13.5 min 2-port vs 29.7 ± 19.9 min 3-port, *P =* .007) No differences in complications, length of hospital stay, or analgesia use	2B	SILC may require longer operative time than 2-port and 3-port LC.
Sulu et al	RCT	Single center	Included elective cholecystectomy Excluded acute cholecystitis, pregnant women, clotting disorders	23				SILC + sub-xiphoid port for fundus, *N* = 23	Postop pain Analgesia Operative time Length of hospital stay	For SILC: Longer operative time (79.1 ± 7.7 vs 35.0 ± 12.3 min, *P <* .05) No differences in postop pain, analgesia, length of hospital stay, or complications	2B	SILC requires much longer operative time than 2-port LC.
Umemura et al	RCT	Single center	Included symptomatic cholelithiasis, previous abdominal surgery Excluded acute cholecystitis with Tokyo grade III, ASA IV, Mirizzi syndrome, choledocholithiasis, suspected malignancy	52				3-port needlescopic (with 12-mm umbilical trocar), *n* = 53	Postop pain at 24 h Analgesia Operative time Length of hospital stay Cosmesis at 3 and 14 d	For SILC: Higher postop pain at 24 h (3.0 ± 2.0 vs 2.1 ± 1.4, *P =* .009) More analgesia doses required (0.8 ± 0.6 vs 1.9 ± 1.3, *P =* .003) No differences found in operative time, length of stay, complications, or cosmesis	2A	MLC may result in less postoperative pain and analgesia use than SILC.
Tamini et al	Systematic review	13 RCTs, 30 obs	RCTs and observational studies comparing SILC versus standard multiport (3 or 4 trocars)	513 RCT, 1577 obs				477 RCT, 4912 obs 3- or 4-port standard	Postop pain at 24 h Operative time Length of stay Return to baseline Cosmesis	For SILC: Less postop pain (*P <* .0001) Longer operative time (*P <* .0001) Shorter length of hospital stay (*P <* .0001) Quicker return to baseline (*P <* .0001) Improved cosmesis (*P <* .0001)	2A	SILC has no greater safety risk than 4-port LC. Operative times may be increased with SILC, but patients may experience less pain, quicker return to baseline, and improved cosmesis.
Tan et al	Systematic review	4 RCTs, 2 obs	RCTs and observational studies comparing SILC and MLC	120 RCT, 558 obs			120 RCT, 1966 obs		Postop pain Analgesia Operative time Length of stay Cosmesis	For SILC: Longer operative time (mean difference 10.67 min, *P =* .007) Complications and conversions skewed higher for SILC but did not reach significance No differences in length of hospital stay, analgesia, or cosmesis	2A	SILC involves longer operative times than MLC without noticeable differences in cosmesis, pain, or length of stay.

None of the RCTs reported significantly increased rates of complications for SILC. Of the 3 systematic reviews, Tamini et al noted no increase in complications [38], whereas Tan et al and Allemann et al noticed slightly elevated though insignificant complications with SILC [31,39]. The systematic review of Allemann et al specifically evaluated bile duct injury (BDI) and other biliary complications (requiring readmission or intervention such as endoscopic retrograde cholangiopancreatography [ERCP] or drain placement) after SILC versus 4-LC, but even their study was underpowered [31]. Their power calculations revealed that with their combined BDI rate of 0.7%, 14,048 patients would be needed to detect a difference in BDI; thus, all included studies were underpowered to detect these relatively rare complications. The main benefit of SILC is improved cosmesis at the cost of longer operative time. Postoperative pain is not reliably reduced. Perhaps partially because of the rarity of biliary complications, no studies showed increased complication rate with SILC. Complications with SILC remain acceptably low, suggesting that SILC technique remains an option for LC.

#### PICO 1 Overall Recommendation

2-LC and 3-LC resulted in quicker postoperative return to baseline without increased operative time but infrequently yielded improvements in cosmesis. Similarly, MLC yielded reduced postoperative pain compared with 4-LC without differences in cosmesis or operative time. Although SILC was associated with longer operative times, this option demonstrated more consistent cosmetic benefit. There were no significant differences in complication rates, although even the meta-analyses may be underpowered to compare complication rates. Benefits of mildly reduced postoperative pain, quicker return to activity, and cosmesis may be weighed against surgeon skill/preference and accordingly longer operative times. All studied port configurations are comparable from a safety and perioperative morbidity standpoint and thus remain acceptable choices per surgeon preference, which the SAGES guideline noted as well [3], although EASL and Tokyo guidelines both advocated against reduced-port LC in the absence of affirmative evidence of benefit [40,41]. Reduced-port and SILC should be considered a safe option only by those with significant training in these techniques. Notably, no included studies evaluated the impact of port placement on achievement of the critical view of safety, which was emphasized in the recent SAGES consensus statement as the most important element of safe LC (see PICO 2) [5]. Future studies regarding optimal port placement should collect this information as proxy for relative safety of various techniques.

**PICO 2: In adult patients undergoing laparoscopic cholecystectomy for acute cholecystitis or symptomatic cholelithiasis, what method of identifying the cystic artery and duct is safest?**

### Background

Several methods exist for identifying the cystic duct and artery safely. Fundus-first dissection involves grasping the fundus and dissecting the gallbladder off of the cystic plate from fundus toward the cystic duct and artery [42]. Critical view of safety technique requires dissection of the hepatocystic triangle, bounded by the cystic duct, common hepatic duct, and inferior edge of the liver, and dissection of the lower third of the gallbladder from the cystic plate. Usually, this dissection is initiated at the triangle of Calot (between the cystic duct, common hepatic duct, and cystic artery). Once these steps are complete and only 2 structures are seen entering the gallbladder, the cystic duct and artery may be safely identified and divided [43]. The "infundibular technique" is not commonly used but is a pared down version of critical view of safety, whereby a surgeon merely has to confirm that the cystic duct is in continuity with the infundibulum of the gallbladder prior to dividing it [44]. Dissection techniques may be augmented with intraoperative cholangiography, fluorescent cholangiography, or laparoscopic ultrasonography (LUS), all of which may be used for either elucidation of biliary anatomy or detection of common bile duct (CBD) stones [45,46]. Four RCTs evaluated critical view of safety and dissection methods used to obtain it. Two RCTs and three systematic reviews investigated radiographic, fluorescent, and ultrasound intraoperative bile duct visualization (Table 7).

**Table 7:** PICO 2 studies

*Series*	*Type*	*Setting/Studies*	*Patients/Study inclusion criteria*	*Comparators*	*Outcomes assessed*	*Conclusion*	*Quality of evidence*	*GRADE recommendation*
Cengiz et al	RCT	Single center	Included symptomatic cholelithiasis and acute cholecystitis	Electrocautery dissection from triangle of Calot (*n* = 37) versus fundus-first dissection with ultrasonic shears (*n =* 43)	Postop pain Analgesia Postop nausea Operative time Length of stay Complications	For ultrasonic fundus-first dissection: Shorter operative time (46 vs 61 min, *P <* .001) Fewer patients requiring overnight stay (2 vs 8 patients, *P =* .036) Lower pain at 4 h (0.8 vs 1.6, *P =* .002) and 24 h (1.5 vs 2.6, *P =* .003) Lower postop nausea (0.3 vs 1.2, *P =* .023 at 2 h, 0.3 vs 1.1, *P =* .002 at 4 h, and 0.5 vs 1.7, *P <* .001 at 24 h) No differences in complications or analgesia use	2B	Ultrasonic fundus-first dissection may be faster and may result in decreased postoperative pain and nausea compared to conventional electrocautery dissection from the triangle of Calot.
Saeed et al	RCT	Single center	Included age 20–60 y with symptomatic cholelithiasis, ASA I/II Excluded acute cholecystitis, choledocholithiasis, previous abdominal surgery, suspected malignancy, pregnancy	Fundus-first dissection with ultrasonic shears (*n =* 41) versus conventional dissection at Calot's triangle (*n =* 41)	Operative time Proportion requiring overnight stay	For fundus-first dissection: Shorter operative time (46.44 ± 6.71 min vs 57.61 ± 13.31 min, *P <* .001) Fewer overnight stays (7.3% vs 36.6%, *P =* .001)	2A	Ultrasonic fundus-first dissection results in shorter operative times and fewer overnight stays compared to conventional dissection at Calot's triangle.
Gupta et al	RCT	Single center	Included symptomatic cholelithiasis Excluded choledocholithiasis, bilioenteric fistula, malignancy	Fundus-first dissection with electrocautery (*n =* 45) versus conventional dissection at Calot's triangle (*n =* 99)	Operative time Conversion between methods Length of hospital stay Complications/bile spillage	For fundus-first dissection: Shorter operative times for noninflamed gallbladders (50.2 ± 11.4 vs. 60.95 ± 18.1 min, *P <* .05), longer operative times for inflamed gallbladders (104.8 ± 18.6 vs. 89.8 ± 14.1 min, *P <* .05) Lower rate of crossover (0 vs. 27 patients, *P <* .05) Lower rate of bile spillage (13.3% vs. 21.2%, *P <* .05) For patients with bile spillage: No difference observed in length of hospital stay	2A	Fundus-first dissection may be quicker in patients with noninflamed gallbladders, may reduce the rate of bile spillage, and may be an effective bailout technique in patients for whom triangle of Calot dissection is difficult.
Zarin et al	RCT	Single center	Included symptomatic cholelithiasis, ASA I Excluded previous abdominal surgery	Critical view of safety technique (*n =* 218) versus infundibular technique (*n =* 220)	Operative time BDI	For critical view of safety: Shorter operative time (50 ± 1.5 vs. 73 ± 2.3 min) Fewer major bile leaks (0.5% vs 1.4%) No significant difference in minor bile leaks (0.5% vs 0.9%)	2B	Using the critical view of safety may reduce operative time and decrease CBD injuries in comparison to the infundibular technique.
Slim et al	Systematic review	6 obs	Studies evaluating whether intraoperative cholangiography reduces incidence of BDI (BDI)	Routine intraoperative cholangiography (IOC) versus LC without routine IOC. Total *n =* 1,889,047	BDI	Two of 6 included studies showed reduced risk of BDI with routine IOC (34% and 70%) One study showed reduced mortality risk with routine IOC (62%) Three studies showed no benefit of routine IOC	2B	Routine IOC may reduce rates of biliary complications, although the evidence is not conclusive.
Ford et al	Systematic review	8 RCT	Studies evaluating routine IOC for BDI prevention	Routine or selective intraoperative cholangiography (IOC) versus LC without routine IOC. Total *n =* 1715	Operative time BDI CBD stone detection	For IOC: Longer operative time (16 min average difference, range 10–23 min) 51 CBD stones correctly detected on IOC, 24 false-positive cholangiograms, 13 false-negative cholangiograms No significant difference in BDI incidence (2 BDIs in IOC groups; 2 BDIs in non-IOC groups)	2A	Routine IOC requires longer operative times without appreciable decreases in BDI or CBD stone retention rates; no recommendation offered.
Ding et al	RCT	Single center	Included symptomatic cholelithiasis Excluded suspicion of choledocholithiasis, pancreatitis, intrahepatic duct dilation, malignancy	Routine LC (*n =* 185) and LC with IOC (*n =* 186)	Operative time Length of stay BDI Retained CBD stone	For IOC: Longer operative time (52.86 ± 4.47 vs 43.0 ± 4.15, *P <* .01) No differences in BDI incidence (1 in each group), hospital stay, conversion to open, or CBD stone retention (0 in IOC group vs 1 in control group)		Routine IOC lengthens mean operative time without appreciable decreases in BDI or CBD stone retention rates; no recommendation offered.
Lehrskov et al	RCT	Single center	Included patients undergoing LC with "complicated gallstone disease" (acute cholecystitis, gallstone pancreatitis, cholangitis, choledocholithiasis) with any detected CBD stones removed via ERCP preoperatively	Intraoperative conventional x-ray cholangiography (*n =* 60) vs intraoperative fluorescent cholangiography (*n =* 60)	Visualization rate of CBD, cystic duct, common hepatic duct, and junction of biliary ducts Ease of technique	For conventional x-ray cholangiography: Improved detection of right and left hepatic ducts (51 vs 16 patients, *P <* .001 for both) Greater surgeon-rated difficulty (2.36 ± 1.03 vs 1.90 ± 0.89, *P =* .011) Equivalent performance for critical junction, CBD, cystic duct, and common hepatic duct	2A	Fluorescent cholangiography is a viable alternative to conventional x-ray cholangiography for visualizing extrahepatic biliary structures.
Dili et al	Systematic review	2 meta-analyses, 18 obs	Studies comparing LUS with IOC	LUS versus IOC. Total *n =* 5302	Ability to map biliary anatomy Ability to detect CBD stones Prevention of conversion in difficult LC BDI rate	For LUS: Complete visualization of 92%–100% of extrapancreatic biliary anatomy Complete visualization of 73%–100% of intrapancreatic biliary anatomy (slightly worse performance than IOC in 1 meta-analysis) Prevention of conversion in 91% of patients with difficult anatomy in 1 study CBD stone sensitivity 76%–100%, specificity 96.2%–100%, possibly superior to IOC No usable BDI data reported in included studies	2A	LUS is a viable alternative to intraoperative cholangiography in most cases with equivalent CBD stone detection and extrapancreatic anatomy delineation.

### Critical View of Safety Versus Infundibular Technique

In a single-center comparative study, Zarin et al randomized patients undergoing laparoscopic cholecystectomy to either "infundibular technique" involving only identification of the cystic duct prior to cystic duct division (*n* = 220) or critical view of safety technique (CVS) (*n =* 218) [44]. Major bile leaks were reduced for patients in the CVS group (0.5% vs 1.4%), and operative time was shorter. Rates of minor bile leaks were comparable between both groups (0.5% vs 0.9%). Although others may consider any CBD injury to be a significant surgical event, Zarin et al defined "minor" and "major" bile leaks as varying degrees of CBD injury. Minor bile leak was defined as a < 25% CBD diameter injury, and major leak was > 25% CBD diameter injury or presence of CBD stricture per McMahon et al classification [47]. This study was weakened by the omission of adequate statistical analysis including *P* values or confidence intervals, but since CVS is so widely accepted, this paper was the only direct comparison found between CVS and an alternate criterion for cystic duct and artery division [44].

### Dissection to Obtain the Critical View of Safety

Three RCTs compared fundus-first dissection against initiating dissection near the infundibulum. Cengiz et al and Saeed et al performed fundus-first dissection with ultrasonic shears and used electrocautery for dissection beginning near the infundibulum/triangle of Calot [48,49], whereas Gupta et al used electrocautery for both approaches [50]. Ultrasonic fundus-first dissection led to significantly shorter duration of operation and fewer overnight hospital stays in both studies [48,49], which may be related to postoperative pain and nausea reductions investigated by one of the studies [48]. Neither study was powered to detect differences in BDI. For electrocautery fundus-first dissection, patients had shorter operative times on noninflamed gallbladders but longer operative times on inflamed gallbladders compared to electrocautery dissection beginning near the infundibulum [50]. However, 27 patients randomized to infundibulum-first dissection (23 of whom had inflamed gallbladders) required conversion to fundus-first technique, and 3 of these were converted to open, skewing the fundus-first group toward more difficult and less time-efficient dissections. Bile spillage occurred less frequently in the fundus-first group and mostly among patients with gallbladder inflammation. Although the high crossover rate suggests advantages in fundus-first dissection, it also limits utility of other comparisons drawn from this study. Taken all together, fundus-first approach may yield mild advantages over infundibulum-first approach for both ultrasonic and electrocautery dissection when seeking the critical view of safety.

### Routine Intraoperative Cholangiography

Two systematic reviews and 1 RCT evaluated the role of intraoperative cholangiography (IOC) in preventing or detecting BDI and CBD stones during cholecystectomy. Slim et al, in a systematic review, excluded studies including fewer than 12,000 patients (because of low incidence of BDI) and found that half of included large-scale studies demonstrated a protective effect of routine IOC [51]. The two largest studies showed 34% (0.34% vs 0.48%) [52] and 33% (0.39% vs 0.58%) [53] overall risk reductions for BDI, and another showed 62% reduction in mortality risk (1.1% vs 3.9% 1-year mortality) [54]. In contrast, systematic review by Ford et al did not reveal reductions in BDI with routine or selective IOC but did elucidate longer operative times whenever IOC was performed [55]. In their evaluation, IOC accurately detected 51 CBD stones, with 24 false positives and 1 false negative for a specificity of 68% and sensitivity of 98%. RCT by Ding et al attempted to compare LC with and without routine IOC and, despite recruitment of 371 patients, was limited by low BDI (only 1 in each group) and low postoperative symptomatic CBD stone occurrence preventing conclusions about BDI or CBD stone detection with IOC [56]. IOC did result in longer mean operative time. BDI is quite rare, but even though routine IOC requires longer operative time, there is some evidence to suggest that it may further decrease the already-low rate of BDI in LC.

### Fluorescent Cholangiography and Laparoscopic Ultrasound

In addition to IOC, there are several newer, alternative intraoperative techniques for identifying the extrahepatic biliary system. Lehrskov et al performed an RCT comparing IOC with indocyanine green fluorescent cholangiography in patients with complicated gallstone disease (acute cholecystitis, gallstone pancreatitis, cholangitis with choledocholithiasis) who underwent preoperative ERCP [57]. Although IOC provided improved visualization of the right and left hepatic ducts, no difference was observed between techniques for evaluation of the cystic duct, CBD, junction of biliary ducts, or common hepatic duct. Surgeons rated fluorescent cholangiography as significantly easier than IOC.

Systematic review by Dili et al compared LUS and IOC for prevention of BDI; however, no included studies reported any incidences of BDI [45]. LUS provided complete visualization of extrapancreatic biliary anatomy in 92%–100% of patients and complete visualization of intrapancreatic biliary anatomy slightly less often (73%–100%). CBD stone sensitivity and specificity were noted to be superior to IOC in one included meta-analysis (0.90 and 0.99 for LUS and 0.87 and 0.98 for IOC, *P* < .05 for both) and comparable to IOC in the other included meta-analysis. The authors note that qualitative advantages of LUS include avoidance of radiation and ability to use LUS before dissection of Calot's triangle, which is not possible with traditional x-ray IOC. Fluorescent cholangiography and LUS yield some advantages over IOC (ease of use, reduction of radiation) and provide reliable information about extrahepatic biliary anatomy; however, these newer techniques have not yet been shown to reduce incidence of BDI.

#### PICO 2 Overall Recommendation

Critical view of safety is the standard method of cystic duct and artery identification supported by surgical society guidelines [3,5,41,43,58], which may explain the paucity of studies comparing CVS to other techniques. In SAGES's recent consensus Delphi study on factors contributing to safe LC, obtaining the critical view was rated as the most important element [5]. Only one RCT compared the CVS with the infundibular technique (less comprehensive dissection prior to cystic duct division), finding that CVS is superior [44]. To obtain this critical view of safety and correctly identify the cystic duct and artery, fundus-first dissection may provide advantages over beginning dissection lateral and medial to the triangle of Calot. Fundus-first dissection is also sometimes useful as a bailout maneuver when dissection in the triangle of Calot is difficult provided the CVS can be conserved, although SC may also be an option in this situation (see PICO 4). SAGES guideline provides both fundus-first and infundibulum-first dissection as first-line options, per surgeon preference [3], whereas WSES [58] and Tokyo [41] guidelines consider fundus-first dissection to be an acceptable bailout maneuver.

Traditional x-ray IOC is sometimes used as an adjunct to elucidate biliary anatomy or identify CBD stones, but because BDI is already quite rare, routine IOC for possible small reductions in BDI may not be worth the increased operative time it takes to perform. SAGES guideline agrees on this point, maintaining that routine IOC reduces BDI, but a selective approach may be more efficient once guidelines for selective IOC are established [3]. EASL and WSES guidelines both contend that for patients at low risk of CBD stones, IOC is not warranted [40,58]. Intraoperative ultrasound and intraoperative fluorescent cholangiography are gaining favor [ 58], providing valuable adjunctive information on biliary structure anatomy [59] without the need to predissect the triangle of Calot or expose the patient to radiation. SAGES supports use of intraoperative ultrasound in certain scenarios [3], and WSES advocates for use of fluorescent cholangiography [ 58]. However, these newer modalities have not yet been shown to reduce incidence of BDI.

**PICO 3: In adult patients undergoing laparoscopic cholecystectomy for acute cholecystitis or symptomatic cholelithiasis, what method of dividing the cystic artery and duct is safest?**

### Background

Once the cystic duct and artery have been identified and dissected, several options exist for division. Laparoscopic application of titanium or nonabsorbable polymer clips is common because of reliability of method and ease of use. However, occasional cases of clip migration have resulted in complications [60], prompting some to advocate for clipless ligation of the cystic artery and duct. Two RCTs and 1 systematic review were found investigating various methods of cystic artery and duct division (Table 8). Our search returned no prospective studies or systematic reviews evaluating stapled transection of the cystic duct or artery, although retrospective evidence supports use of a laparoscopic stapler for dilated or difficult cystic ducts [61].

**Table 8:** PICO 3 studies

*Series*	*Type*	*Patients/Study inclusion criteria*	*Comparators*	*Outcomes assessed*	*Conclusion*	*Quality of evidence*	*GRADE recommendation*
Baloch et al	RCT (single center)	Included symptomatic cholelithiasis Excluded previous abdominal surgery and conversions to open	Cystic duct/artery ligation with titanium clips (*n =* 41) versus harmonic shears (*n =* 40)	Operative time Complications	For harmonic shears: Shorter operative time (21.5 vs 26.6 min, *P =* .002) No differences in bleeding events or bile leaks	2B	Harmonic shear division of the cystic artery and duct results in shorter operative time, with unknown impact on complication rates
Sanawan et al	RCT (single center)	Included symptomatic cholelithiasis Excluded previous abdominal surgery, choledocholithiasis, evidence of obstructive jaundice	Cystic duct/artery ligation with titanium clips (*n =* 75) versus harmonic shears (*n =* 75)	Operative time Complications Bile leak/subhepatic fluid collection at 2 and 4 wk	For harmonic shears: Shorter operative time (30 vs 35 min, *P <* .0001) Fewer gallbladder perforations (5 patients, 6.7% vs 16 patients, 21.3%; *P =* .01) Fewer instances of liver bed bleeding (1 patient, 1.3% vs 23 patients, 31%; *P <* .0001) No bile leaks on 2- and 4-wk follow-up ultrasound	2B	Clipless ligation of the cystic duct and artery using harmonic shears was quicker and resulted in fewer perioperative complications than standard titanium clip LC with electrocautery dissection.
Dijk et al	Systematic review	4 RCTs, 10 comparative, 24 obs	Metal clips (*n =* 38,683) versus ligature (*n =* 3604) versus locking clips (*n =* 1853) versus harmonic scalpel (*n =* 1692) versus absorbable clip (*n =* 1299) versus LigaSure (*n =* 230)	CDL	CDL after harmonic versus clip division: OR 0.4 (95% CI 0.06–2.48), slightly lower rate after harmonic shears CDL after locking clips and ligatures versus nonlocking clips: OR 0.17 (95% CI 0.03–0.93) CDL rates were ∼ 1% for harmonic shears and nonlocking clips, and ∼ 0% for locking clips and ligatures	2A	Locking clips and ligatures result in slightly lower rates of CDL after cystic duct/artery ligation than nonlocking clips. CDL rates after harmonic division are comparable with those after clip division.

### Titanium Clips Versus Locking Clips/Ligature Versus Harmonic Shears

Two RCTs and 1 systematic review evaluated ligation of the cystic duct and artery using various methods [[62], [63], [64]]. Dijk et al (systematic review and meta-analysis) included 47,491 patients in total and compared titanium clips (nonlocking), locking clips or ligature, and harmonic shears for division of the cystic duct. Their analysis revealed that harmonic energy resulted in slightly lower, though insignificant, postoperative cystic duct leak (CDL) rates than clip closure (odds ratio [OR] 0.4, 95% confidence interval [CI] 0.06–2.48). Locking clips or ligature had lower CDL than nonlocking clips (OR 0.17, 95% CI 0.03–0.93) [64]. In the RCTs comparing titanium clips against harmonic shears, less operative time was required for the harmonic shear groups. Baloch et al demonstrated no differences in complication rates, with 1 bleeding complication in the titanium clip group and 1 minor bile leak (bile observed in surgical drain at 24 hours, which quickly resolved) in each of the groups [62]. Sanawan et al noted that the harmonic shear group sustained fewer gallbladder perforations (5 patients, 7% vs 16 patients, 21%; *P =* .01) and fewer instances of liver bed bleeding (1 patient, 1% vs 23 patients, 31%; *P <* .0001), although the authors did not define what constituted liver bed bleeding. At 2- and 4-week follow-up ultrasound, there were no bile leaks or subhepatic fluid collection in either group [63]. Neither study distinguished whether benefits of harmonic shear use are due specifically to lack of clip use or harmonic versus electrocautery dissection of the cystic plate. With so few instances of bile leaks at follow-up, these studies could not conclusively distinguish whether clipless ligation is as durable as clip ligation in keeping critical structures closed.

#### PICO 3 Overall Recommendation

Division of cystic duct with a locking clip or ligature may result in lower rates of CDL than nonlocking clip, supporting the preference of locking clips when feasible from a cost perspective. Clipless cystic artery and duct ligation with harmonic shears seems to result in quicker operative time compared to traditional clip ligation without a rise in intraoperative complications or increased risk of CDL. However, none of the included studies adequately evaluated long-term risk of clip migration because it is a rare complication. Additionally, no included study controlled for gallbladder dissection technique: when harmonic scalpel was used to divide the cystic duct/artery, it may have also been employed for dissection, confounding findings. Although clipless ligation has not been demonstrated to be inferior to clip ligation, it cannot yet be recommended over clip ligation. No commonly used society guideline commented on technique for division of cystic duct and artery.

**PICO 4: In adult patients undergoing difficult laparoscopic cholecystectomy, when and how should SC be performed?**

### Background

Subtotal cholecystectomy, which constitutes removal of portions of the gallbladder, is performed in difficult cholecystectomies where inflammation or adhesions in Calot's triangle preclude safe dissection to facilitate obtaining the critical view of safety and usual ligation of the cystic duct and artery [65]. Alternative approaches to SC include open cholecystectomy, fundus-first dissection, and cholecystostomy tube placement to allow the gallbladder to decompress, facilitating cholecystectomy at a later time [42,66,67]. SC can be fenestrated, with gallbladder stump left open and drain placed with or without internal closure of the cystic duct, or reconstituted, with staples placed across the infundibulum. Fenestrated SC may put patients at risk for postoperative fistula, whereas reconstituted SC may put patients at risk of recurrent symptomatic cholelithiasis from stone reformation in the remnant pouch [68]. Three systematic reviews investigate predictors, indications, and outcomes of SC (Table 9).

**Table 9:** PICO 4 studies

*Series*	*Type*	*Studies included*	*Comparators*	*Outcomes assessed*	*Conclusion*	*Quality of evidence*	*GRADE recommendation*
Elshaer et al	Systematic review	30 RCT and obs	Laparoscopic SC (*n =* 898), open SC (*n =* 234), laparoscopic converted to open SC (*n =* 99)	Indications for SC Complications Reoperations Postop ERCP Mortality	Indications: inflammation (72.1%), cirrhosis (18.2%), perforation/empyema (6.1%), Mirizzi (3.0%) Complications: bile leaks (18.0%) postoperative hemorrhage (0.3%), subhepatic collection (2.9%), BDI (0.08%), wound infection (2.6%), and retained stones (3.1%) For open cystic duct/GB stump: more bile leaks (42.0% vs 16.5%) and more retained stones (12.0% vs 2.4%) Reoperation (1.8%): CBD exploration for stones (22.7%), abscess/fluid collection (22.7%), completion LC (18.2%) 30-d mortality: 0.4%	2A	SC is a viable bailout technique with a higher rate of bile leak and retained stones when the fenestrated technique is used.
Henneman et al	Systematic review	15 Obs	A: posterior wall remains, open stump (*n =* 332) B: posterior wall remains, closed stump (*n =* 24) C: posterior wall resected, closed stump (*n =* 200) D: posterior wall resected, open stump with drain (*n =* 60)	Conversion rate Bile leak Recurrent gallstones Percutaneous intervention Reoperation Postop ERCP	For group D: highest rates of conversion (50%), ERCP (10%), percutaneous intervention (5%) For group A: highest rates of bile leak (16%), recurrent gallstones (1.8%), reoperation (4.7%)	2B	Stump closure in SC is associated with lower rates of bile leak and the need for reinterventions.
Hussain et al	Systematic review	91 studies: 3 meta-analyses, 5 RCTs, 21 prospective obs, 63 retrospective obs	Difficult versus nondifficult LC. Total *n =* 324,553.	Predictors of difficult LC Conversion rate	Predictors of difficult LC: male sex, greater age, obesity, cirrhosis, adhesions, emergency cholecystectomy, acute cholecystitis, cystic duct stones, large liver and gallbladder For difficult dissection of Calot's triangle (inflammation/anatomy) when laparoscopic SC is used, conversion to open rate is 0.5%.		Insufficient data to issue a recommendation—evaluated risk factors but did not compare techniques.

### Indications and Predictors of Difficult Cholecystectomy

The systematic review of Hussain et al of 91 studies on difficult cholecystectomy included 324,553 patients [69]. Male sex, older age, obesity, cirrhosis, adhesions, emergency cholecystectomy, acute cholecystitis, cystic duct stones, and large liver and gallbladder were associated with a more difficult operation. Elshaer et al (a systematic review including 1,231 patients) noted that indications for SC were severe inflammation at Calot's triangle (72%), cirrhosis and portal hypertension (18%), perforation and empyema (6%), and Mirizzi syndrome (3%) [70].

### Outcomes of SC

Hussain et al also evaluated conversion to open for SC and determined that the use of laparoscopic SC kept the conversion rate to 0.5%. The authors report low complication rates in all 12 studies evaluating SC, concluding that it is a safe option, although they do not report a comparator group.

For more granular analysis, both Henneman et al and Elshaer et al assessed complications of laparoscopic SC by operative technique [65]. Each review assessed bile leaks, retained stones, and reoperation across several operative choices: resection of the posterior gallbladder wall versus leaving it on the cystic plate, and closing the gallbladder stump versus leaving it open (fenestrated). In both studies, fenestrated SC was associated with significantly higher rates of bile leaks, recurrent/retained stones, and reoperation. Elshaer et al also calculated mortality for all included patients undergoing SC and noted an overall 30-day mortality of 0.4%. They noted that this is more common than the rate of reoperation and mortality for total cholecystectomy (0.2% and 0.08%, respectively).

#### PICO 4 Overall Recommendation

Subtotal cholecystectomy is indicated in LC where Calot's triangle cannot be safely dissected and the critical view of safety cannot be obtained per Tokyo 2018 guidelines [41], avoiding injury to the bile duct and nearby vascular structures. In addition, it may also reduce conversions to open cholecystectomy. Although SC is associated with more reinterventions and higher mortality than total cholecystectomy, patients requiring SC are usually more ill, confounding results. Somewhat intuitively, SC is thought to prevent BDI in patients with inflamed, complicated gallbladders [41]. Failure to ligate the cystic duct and/or close the gallbladder stump may result in higher rates of postoperative bile leaks [70] and reoperation [65]. Authors noted that drains were left more frequently in fenestrated SC, but no study provided guidance on the decision to leave a drain, and guidelines support a role for drains in complicated LC [3]. There is no evidence to favor SC over other techniques for managing difficult gallbladder disease including fundus-first dissection, percutaneous cholecystostomy, or open cholecystectomy. Notably, no studies that met inclusion criteria compared laparoscopic SC to open total cholecystectomy, although previous work has suggested that laparoscopic SC is associated with lower morbidity and mortality than open cholecystectomy [41,58].

**PICO 5: In adult patients undergoing laparoscopic cholecystectomy for acute cholecystitis or symptomatic cholelithiasis, what are the best practices to extract the gallbladder to minimize perioperative comorbidities including surgical site infection and port site hernia?**

### Background

The ideal method of specimen removal to minimize postoperative pain, port site hernia, and surgical site infection remains unclear [71]. Specimens may be removed from the umbilical or epigastric port sites using either an endocatch bag or a surgical glove, or directly from the body without a container [71,72]. Current SAGES guidelines for biliary surgery do not make recommendations for specimen extraction because of insufficient data [3]. Our literature review revealed 6 studies addressing specimen extraction techniques (Table 10).

**Table 10:** PICO 5 studies

*Series*	*Type*	*Patient criteria/studies included*	*Comparators*	*Outcomes assessed*	*Conclusion*	*Quality of evidence*	*GRADE recommendation*
Kulkarni et al	Systematic review	9 RCT	Epigastric versus umbilical port gallbladder extraction (*n =* 1036)	Postop pain at 24 h Operative time Time to gallbladder removal Port site infection Port site hernia	For umbilical removal: Lower rate of port site hernia (RR 2.68, 7.2% vs 2.2%, *P =* .04) No difference in port site infection (4.3% vs 2.8%, *P =* .93) No difference in postop pain, operative time, or gallbladder retrieval time	2A	Epigastric port gallbladder extraction may be associated with reduced risk of port site hernia without impact on port site infection rates, postop pain, or operative time.
Mongelli et al	Systematic review	7 RCT	Epigastric versus umbilical port gallbladder extraction (*n =* 876)	Postop pain at 1, 6, 12, and 24 h Operative time Port site infection Port site hernia	For umbilical removal: Reduced postop pain at 1 h (mean difference − 1.102, *P* < .001), 6 h (− 1.021, *P* < .001), 12 h (− 1.417, *P* < .001), and 24 h (− 0.447, *P* = .034) No difference in operative time, surgical site infection, or hernia incidence	2A	Umbilical port site removal is associated with reduced postoperative pain in the first postoperative day but does not affect the incidence of port site hernia or surgical site infection.
Sood et al	Systematic review	7 RCT, 1 obs	Epigastric versus umbilical port gallbladder extraction (*n =* 2676)	Postop pain at 24 h Gallbladder perforation rate Gallbladder retrieval time Gallbladder retrieval difficulty Operative time Port site infection Port site hernia	For umbilical removal: Longer operative time (MD 0.41, *P =* .004) Less postop pain (MD − 0.51, *P =* .03) Lower perforation rate (OR 0.37, *P* = .002) Lower retrieval time (MD − 0.43, *P =* .008) Less frequent gallbladder retrieval difficulty (OR 0.34, *P* = .0008) No difference in port site infection or port site hernia	2B	Umbilical site removal is associated with longer operative time but reduced pain at 24 h and easier gallbladder retrieval
Hajibandeh et al	Systematic review	5 RCTs, 1 obs	Epigastric versus umbilical port gallbladder extraction (*n =* 2394)	Postop pain at 24 h Gallbladder retrieval time Port site infection Port site hernia	For umbilical removal: Shorter retrieval time (MD − 1.83 min, *P =* .008) No difference in postop pain, port site infection, or port site hernia	2B	Gallbladder removal through the umbilical port is associated with reduced retrieval time.
La Regina et al	Systematic review	2 RCTs, 1 obs	Gallbladder retrieval bag versus no bag (*n =* 605)	Wound infections	Wound infections were slightly less common when retrieval bag was used (4.2% vs 5.9%, RR 0.82, 95% CI 0.41–1.63), but difference was not significant.	2B	Gallbladder specimen retrieval bags are not associated with decreased surgical site infection.
Rehman et al	RCT (single center)	Included symptomatic cholelithiasis, age 25–60 y Excluded acute cholecystitis, empyema, liver enzyme derangements	Gallbladder retrieval bag (*n =* 127) versus no bag (*n =* 127)	Wound infections	Wound infections were less common with retrieval bag (1 patient, 0.4% vs 14 patients, 5.5%, but no *P* value provided)	2B	Gallbladder specimen retrieval bags may reduce the risk of surgical site infection.

### Port Site—Umbilical Versus Epigastric

Four systematic reviews compared patient outcomes after gallbladder extraction from the epigastric or umbilical port [71,[73], [74], [75]]. Only 1 study, Kulkarni et al observed differences in port site hernia, with more frequent occurrence in the umbilical port gallbladder extraction group over a follow-up of 30 days to 6 months [71]. No studies noted a difference in surgical site infection after extraction from epigastric or umbilical incision.

Mongelli et al and Sood et al both observed that patients undergoing umbilical port site removal had less immediate postoperative pain in the day following surgery [73,74], whereas Kulkarni and Hajibandeh found no difference in postoperative pain [71,75]. Furthermore, Hajibandeh and Sood noted significantly quicker gallbladder retrieval time from the umbilical site, with the latter describing fewer instances of gallbladder perforation during the extraction [74,75]. Umbilical retrieval seems to be associated with easier, less traumatic gallbladder extraction.

Both Sood and Hajibandeh's systematic reviews may be subject to selection bias due to the relatively high number of patients (*n =* 1800) included from the same nonrandomized study, contributing > 50% of the patients in each [76].

### Direct Specimen Removal Versus Specimen Retrieval Bag

One systematic review (La Regina et al*)* and 1 RCT (Rehman et al) compared surgical site infections with and without use of a gallbladder retrieval bag [72,77]. La Regina observed no difference in incidence of wound infection rate between the retrieval bag (4%) and direct retrieval groups (6%) (risk ratio [RR] 0.82, 95% CI 0.41–1.63), although the incidence of surgical site infection in the included studies was higher than the Centers for Disease Control and Prevention's acceptable rate (1.6%–3.2%), which may result from utilizing different criteria. Rehman did find fewer wound infections in the retrieval bag group (1 patient, 0.4% vs 14 patients, 5.5%), but no *P* value was provided. Insufficient statistics reporting casts doubt on the reliability/generalizability of these findings.

#### PICO 5 Overall Recommendations

Only 1 out of 4 systematic reviews comparing hernia risk by gallbladder extraction site found an association between umbilical extraction and hernia, whereas the remaining 3 reviews found evidence that umbilical extraction resulted in less pain and/or easier and quicker extraction with less risk of gallbladder perforation. It must be noted that regardless of extraction location, the incision must be made large enough relative to the largest gallstone to facilitate successful extraction. No studies demonstrated a difference in surgical site infections. Benefit of the use of a gallbladder retrieval bag was equivocal, with one lower-quality RCT showing possible surgical site infection reduction and the other systematic review showing no difference. Similarly, SAGES guideline ruled that there are no data to guide choice of extraction technique [3]. In the absence of strong, consistent evidence supporting umbilical or epigastric extraction either with or without a retrieval bag, surgeon and patient preference should guide choice of gallbladder retrieval method.

## CONCLUSION

Laparoscopic cholecystectomy is a widely performed procedure for which there exists ambiguity in a number of operative choices. By reviewing the evidence evaluating each operative step, we sought to determine whether recommendations could be issued to optimize providers' surgical decision-making and improve patient outcomes. Aside from recommendations on division of the cystic duct and artery, our findings are generally in line with SAGES guideline recommendations from 12 years prior, highlighting the durability of their suggestions in modern practice. Table 11 summarizes our recommendations for each question.

**Table 11:** Summary of recommendations

*PICO question*	*Recommendation*
1: In adult patients undergoing laparoscopic cholecystectomy (LC) for acute cholecystitis or symptomatic cholelithiasis, what is the best configuration of ports to limit perioperative morbidity (including port site hernia) and optimize surgical efficiency?	2-LC and 3-LC may result in quicker postoperative return to baseline. MLC is associated with pain reduction, but instruments may experience technical issues. SILC often yields improved cosmetic satisfaction but may require longer operative time. Because no differences in safety/complications were observed between any technique, all remain acceptable options.
2: In adult patients undergoing laparoscopic cholecystectomy for acute cholecystitis or symptomatic cholelithiasis, what method of identifying the cystic artery and duct is the safest?	Critical view of safety in Calot's triangle should be obtained to minimize risk of BDI, but fundus-first dissection is an acceptable method of dissection to obtain the critical view. Intraoperative ultrasound, intraoperative fluorescent cholangiography, and intraoperative x-ray cholangiography may be helpful aids in elucidating biliary anatomy but are not shown to prevent BDI.
3: In adult patients undergoing laparoscopic cholecystectomy for acute cholecystitis or symptomatic cholelithiasis, what method of dividing the cystic artery and duct is the safest?	Use of locking clips or ligatures may yield marginally lower rates of CDL than nonlocking clips. Although harmonic ligation has not been shown to have higher leak rates than clip ligation, there is insufficient evidence to support the use of clipless ligation in specific situations.
4: In adult patients undergoing laparoscopic cholecystectomy for acute cholecystitis or cholelithiasis, when is an SC indicated?	SC is a valid bailout method when inflammation or anatomy prevents attainment of the critical view of safety. Ligation of the cystic duct/gallbladder stump is associated with fewer bile leaks.
5: In adult patients undergoing laparoscopic cholecystectomy for acute cholecystitis or symptomatic cholelithiasis, what are the best practices to extract the gallbladder to minimize perioperative comorbidities including surgical site infection and port site hernia?	Insufficient evidence to support epigastric versus umbilical site gallbladder extraction. Reductions in surgical site infection from using gallbladder retrieval bag were very modest; routine gallbladder retrieval bag use cannot be recommended on the basis of this evidence.

## Author Contribution

Andrea Fisher: Conceptualization, Methodology, Data curation, Writing – original draft, and Writing – review & editing

Kovi Bessoff: Conceptualization, Methodology, Writing – original draft, and Writing – review & editing

Gavin Touponse: Data curation, Writing – original draft

Rida Khan: Data curation, Writing – original draft

Maggie Yu: Data curation, Writing – original draft

Advait Patil: Data curation, Writing – original draft

Jeff Choi: Conceptualization, Methodology, Writing – review & editing

Christopher Stave: Methodology, Software, Resources

Joseph Forrester: Supervision, Writing – review & editing

## Funding Source

No funding or financial support was received for this work.

## Ethics Approval Statement

This work was granted an exception by our university's Institutional Review Board, as the work does not include any human or animal subjects and instead relies on publicly available, deidentified data.

## Conflict of Interest

No conflicts of interest are reported for any author. Dr Forrester has received unrestricted research funding from Varian for an investigator-initiated clinical trial (https://clinicaltrials.gov/ct2/show/NCT04482582) and received grant funding from the Surgical Infections Society. Neither of these leads to conflicts of interest for this work product.

## Appendix A Supplementary data

Search strategies for PubMed, Embase, and Web of Science. Image 1
