# Supplementary material for: Evidence-based surgery for laparoscopic cholecystectomy
Source: Surg Open Sci. 2022 Aug 18;10:116–34. doi: 10.1016/j.sopen.2022.08.003 (PMC9483801; doi:10.1016/j.sopen.2022.08.003)
Supplement: Supplementary file 1 — Search strategies for PubMed, Embase, and Web of Science. [file mmc1.docx]

**Appendix 1: Search Strategies**

**Port Placement:**

**PICO 1:** In adult patients undergoing laparoscopic cholecystectomy for acute cholecystitis or symptomatic cholelithiasis, biliary colic what is the best configuration of ports to limit perioperative morbidity (including port site hernia) and optimize surgical efficiency?

**PUBMED:**

('laparoscopic cholecystectomy'/exp OR "Laparoscopic cholecystectom*" [tw]) AND ("Cholecystitis, Acute"[Mesh] OR "acute cholecystitis" [tw] OR "Cholelithiasis"[Mesh] OR "Cholelithiasis"[tw]) AND (port [tw] OR ports [tw]) AND english [lang] NOT ("animals" [mesh] NOT "humans" [mesh]) NOT "case reports" [pt] NOT comment [pt]

291 results

**EMBASE:**

('laparoscopic cholecystectomy'/exp OR 'laparoscopic cholecystectom*':ti,ab,kw) AND ('acute cholecystitis'/exp OR 'acute cholecystitis':ti,ab,kw OR 'cholelithiasis'/exp OR cholelithiasis:ti,kw,ab) AND (port:ti,ab,kw OR ports:ti,ab,kw) NOT ('letter'/exp OR 'case report'/exp) AND [humans]/lim AND [english]/lim AND [embase]/lim NOT ([embase]/lim AND [medline]/lim)

382 results

**WOS:**

(TS=("laparoscopic cholecystectom*") AND TI=("acute cholecystitis" OR cholelithiasis)) AND TS=(port OR ports) AND (LA=english)

28 results

**Identification and division of cystic duct and artery:**

In adult patients undergoing laparoscopic cholecystectomy for acute cholecystitis or symptomatic cholelithiasis, what method of identifying and clipping the cystic artery and duct is the safest?

**PUBMED:**

("Cholecystectomy, Laparoscopic"[Mesh] OR "Laparoscopic cholecystectom*" [tw]) AND ("Cholecystitis, Acute"[Mesh] OR "acute cholecystitis" [tw] OR "Cholelithiasis"[Mesh] OR "Cholelithiasis" [tw]) AND  ((identifi* [ti] OR “critical view of safety” [tw] OR infundibular [tw] OR “top down” [tw] OR “fundus first” [tw]) OR ((“cystic duct” [mesh] OR “cystic duct*” [tw] OR “common bile duct” [mesh] OR “bile duct*” [tw] OR “biliary tree” [tw]) AND (“cystic duct/blood supply” [mesh] OR “cystic artery” [tw] OR (cystic [ti] AND arter* [ti])))) AND english [lang] NOT ("animals" [mesh] NOT "humans" [mesh]) NOT "case reports" [pt] NOT comment [pt]

102 results

**EMBASE:**

(("laparoscopic cholecystectomy"/exp OR "laparoscopic cholecystectom*":ti,ab,kw) AND ("acute cholecystitis"/exp OR "acute cholecystitis":ti,ab,kw OR "cholelithiasis"/exp OR cholelithiasis:ti,kw,ab)) AND ((identifi*:ti OR “critical view of safety”:ti,ab,kw OR infundibular:ti,ab,kw OR “top down”:ti,ab,kw OR “fundus first”:ti,ab,kw) OR (("cystic duct"/exp OR "common bile duct"/exp OR “cystic duct*”:ti,ab,kw  OR “bile duct*”:ti,ab,kw OR “biliary tree”:ti,ab,kw) AND ("cystic artery"/exp OR "cystic artery":ti,ab,kw OR "artery clipping":ti,ab,kw OR (cystic:ti AND arter*:ti)))) NOT ("letter"/exp OR "case report"/exp) AND [humans]/lim AND [english]/lim AND [embase]/lim NOT ([embase]/lim AND [medline]/lim)

131 results

**WOS**

(TS=("laparoscopic cholecystectom*") AND TS=("acute cholecystitis" OR cholelithiasis))  AND ((TI=identifi* OR TS=“critical view of safety” OR TS=infundibular OR TS=“top down” OR TS=“fundus first”) OR (TS=“cystic duct*” OR TS=“bile duct*” OR TS=“biliary tree”) AND (TS=”cystic artery” OR TS="artery clip*")) AND (LA=english)

95 results

**Cholangiography:**

In adult patients undergoing laparoscopic cholecystectomy for acute cholecystitis or cholelithiasis, does use of intraoperative cholangiogram or fluorescence cholangiography reduce the risk of common bile duct injury?

**PUBMED:**

("Cholecystectomy, Laparoscopic"[Mesh] OR “Laparoscopic cholecystectom*” [tw]) AND ("Cholecystitis, Acute"[Mesh] OR “acute cholecystitis” [tw] OR "Cholelithiasis"[Mesh] OR "Cholelithiasis"[tw]) AND ("Fluoroscopy"[Mesh] OR "Fluorescent Dyes"[Mesh] OR "Fluorescence"[Mesh] OR fluorescen* [tw] OR "Cholangiography"[Mesh] OR cholangiogram* [tw] OR cholangiograph* [tw]) AND ("intraoperative care" [mesh] OR “intraoperative period” [mesh] OR  intraoperat* [tw]) AND english [lang] NOT ("animals" [mesh] NOT "humans" [mesh]) NOT "case reports" [pt] NOT comment [pt]

716 results

**EMBASE:**

(("laparoscopic cholecystectomy"/exp OR "laparoscopic cholecystectom*":ti,ab,kw) AND ("acute cholecystitis"/exp OR "acute cholecystitis":ti,ab,kw OR "cholelithiasis"/exp OR cholelithiasis:ti,kw,ab))  AND ('fluoroscopy'/exp OR 'fluorescent dye'/exp OR 'fluorescence'/exp OR 'cholangiography'/exp OR fluorescen*:ti,ab,kw OR cholangiogram*:ti,ab,kw OR cholangiograph*:ti,ab,kw) AND ('peroperative care'/exp OR  intraoperat*:ti,ab,kw OR peroperat*:ti,kw,ab) NOT ("letter"/exp OR "case report"/exp) AND [humans]/lim AND [english]/lim AND [embase]/lim NOT ([embase]/lim AND [medline]/lim)

318 results

**WOS**

(TS=("laparoscopic cholecystectom*") AND TS=("acute cholecystitis" OR cholelithiasis))  AND (TS=fluoros* OR TS=cholangiograp* OR TS=cholangiogram*) AND TS=(peroperativ* OR intraoper*) AND (LA=english)

264 results

**Subtotal Cholecystectomy:**

In adult patients undergoing laparoscopic cholecystectomy for acute cholecystitis or cholelithiasis, when is a subtotal cholecystectomy indicated?

**PUBMED**:

("Cholecystectomy, Laparoscopic"[Mesh] OR “Laparoscopic cholecystectom*” [tw]) AND ("Cholecystitis, Acute"[Mesh] OR “acute cholecystitis” [tw] OR "Cholelithiasis"[Mesh] OR "Cholelithiasis"[tw]) AND (“cystic duct” [mesh] OR “cystic duct*” [tw] OR “common bile duct” [mesh] OR “bile duct*” [tw] OR “biliary tree” [tw]) AND (subtotal [tw] OR "cystic plate" [tw] OR "difficult dissection" [tw] OR complic* [ti] OR difficult* [ti] OR severe [ti] OR "conversion to open surgery" [mesh]) AND english [lang] NOT ("animals" [mesh] NOT "humans" [mesh]) NOT "case reports" [pt] NOT comment [pt]

179 results

**EMBASE**:

("laparoscopic cholecystectomy"/exp OR "laparoscopic cholecystectom*":ti,ab,kw) AND ("acute cholecystitis"/exp OR "acute cholecystitis":ti,ab,kw OR "cholelithiasis"/exp OR cholelithiasis:ti,kw,ab) AND ("cystic duct"/exp OR "common bile duct"/exp OR “cystic duct*”:ti,ab,kw  OR “bile duct*”:ti,ab,kw OR “biliary tree”:ti,ab,kw) AND (subtotal:ti,ab,kw OR "cystic plate":ti,ab,kw OR "difficult dissection":ti,ab,kw OR complic*:ti OR difficult*:ti OR severe:ti OR 'conversion to open surgery'/exp) NOT ("letter"/exp OR "case report"/exp) AND [humans]/lim AND [english]/lim AND [embase]/lim NOT ([embase]/lim AND [medline]/lim)

187 results

**WOS:**

(TS=("laparoscopic cholecystectom*") AND TS=("acute cholecystitis" OR cholelithiasis))  AND  TS=(“cystic duct*” OR “bile duct*” OR “biliary tree”) AND (TS=subtotal OR TS="cystic plate" OR TS="difficult dissection" OR TI=complic* OR TI=difficult* OR TI=sever*) AND (LA=english)

180 results

**Specimen extraction:**

In adult patients undergoing laparoscopic cholecystectomy for acute cholecystitis or symptomatic cholelithiasis, what are the best practices to extract the gallbladder in order to minimize perioperative comorbidities including surgical site infection and port site hernia?

**PUBMED:**

("Cholecystectomy, Laparoscopic"[Mesh] OR “Laparoscopic cholecystectom*” [ti]) AND ("endocatch" [tw] OR "endo catch"[tw] OR "endo bag" [tw] OR "endobag" [tw] OR "gallbladder extract*" [tw] OR "gallbladder retriev*" [tw] OR "retrieval bag" [tw]) AND english [lang] NOT ("animals" [mesh] NOT "humans" [mesh])

224 results

**EMBASE:**

("laparoscopic cholecystectomy"/exp OR "laparoscopic cholecystectom*":ti) AND ((gallbladder* NEAR/3 (retriev* OR extract*)):ti,ab,kw OR (endocatch:ti,ab,kw OR “endo catch”:ti,kw,ab OR “endo bag”:ti,ab,kw OR endobag:ti,kw,ab)) NOT ("letter"/exp OR "case report"/exp) AND [humans]/lim AND [english]/lim

101 results

**WOS:**

(TI=("laparoscopic cholecystectom*")) AND (TS = (gallbladder NEAR/3 (extract* OR retriev*))) AND (LA=english)
